# Minimize the extent and morbidity of axillary dissection for node-positive breast cancer patients: implementation of axillary lymph node dissection based on breast lymphatics level

**DOI:** 10.1186/s12885-021-08024-y

**Published:** 2021-03-19

**Authors:** Qianqian Yuan, Jinxuan Hou, Yukun He, Yiqian Liao, Lewei Zheng, Gaosong Wu

**Affiliations:** Department of Thyroid and Breast Surgery, Zhongnan Hospital of Wuhan University, 169 Donghu Road, Wuhan, 430071 Hubei People’s Republic of China

**Keywords:** Breast cancer, Axillary lymph node dissection, Breast cancer related lymphedema

## Abstract

**Background:**

Breast cancer-related lymphedema (BCRL) is associated with extensive axillary dissection. Axillary lymph node dissection (ALND) based on breast lymphatics level (BLL) was proposed to minimize the surgical extent for node-positive breast cancer patients.

**Methods:**

A total of 156 consecutive sentinel lymph node-positive (SLN+) or clinically node-positive (cN+) patients underwent sentinel lymph node biopsy (SLNB) with indocyanine green and methylene blue (MB). The SLNs were injected with 0.1 ml MB before removal, and a standard ALND was subsequently performed. The nodes adjacent to the blue-stained axillary lymph nodes from the breast (bALNs) were sent for pathological examination separately by resecting serial tissue every 0.5 cm away from the marginal blue-stained bALNs. Then, a pilot study comparing ALND based on BLL and standard ALND was performed.

**Results:**

BLL were successfully identified in 20 SLN+ (100%) and 134 cN+ (98.5%) patients. The median number of BLL was four, ranging from three to six. A horizontal line 1.0 cm away from the superior blue-stained bALN and a vertical line 1.0 cm away from the medial blue-stained bALN formed BLL II, III, and IV. All of the additional positive nodes were within 1.0 cm of the blue-stained bALNs. The minimized axillary dissection should resect upwards from the lowest BLL that contains the first confirmed negative blue-stained bALNs. In the pilot study, no patient developed axillary recurrence.

**Conclusion:**

The ALND surgical procedure based on BLL could minimize the surgical extent for pathological node-positive breast cancer patients and potentially reduce the BCRL rate.

**Trial registration:**

ChiCTR1800014247.

**Supplementary Information:**

The online version contains supplementary material available at 10.1186/s12885-021-08024-y.

## Background

The advent of sentinel lymph node biopsy (SLNB) has revolutionized the surgical management of axilla in breast cancer patients [1]. Complete axillary lymph node dissection (ALND) has been gradually replaced for select patients, owing to the substantial morbidity such as debilitating breast cancer-related lymphedema (BCRL), shoulder dysfunction, paresthesia and discomfort [2]. Damage to the lymphatic drainage system in the axilla by multidisciplinary treatment contributes to the occurrence of BCRL [3]. Notably, extensive axillary dissection is related to a high occurrence rate of BCRL. A total of 77.8% of the patients who underwent ALND had fewer than three additional involved nodes in the ACOSOG Z0011 trial [4]. Thus, the complete removal all axillary lymph nodes with ALND for those patients might be over-treatment. For patients who were not eligible for the ACOSOG Z0011 study criteria, especially clinically node-positive breast cancer patients, a method for de-escalating the surgical area of ALND would significantly reduce the BCRL rate.

The lymphatic system of the breast forms an extensive and complex network of periductal and perilobular vessels that drain principally to the axillary nodes [5]. Breast tumors commonly invade local structures and spread in a progressive and sequential manner to regional nodes, and the lymphatic vessels provide anatomical continuity for this process by acting as a link between the primary tumor and the regional nodes. Hence, the sentinel lymph node (SLN) hypothesis presupposes an orderly spread of cancer cells from the primary tumor to the first draining node, which has prevented clinically node-negative breast cancer patients from undergoing extensive axillary dissection [6].

On the theoretical basis of SLNB, for node-positive breast cancer patients, metastasis from breast tumors does not involve the axillary lymph nodes from the breast (bALNs) as a unit but instead progresses from the primary tumor to the first draining nodes, then second and third echelon nodes [7, 8]. Therefore, our institution implemented the approach of ALND based on the lymphatic drainage from the breast in order to limit surgical resection in the axilla and reduce the morbidity of ALND. The primary aim was to accurately determine the extent of every breast lymphatics level (BLL). The secondary aim was to determine the skip metastasis rate to demonstrate the de-escalation feasibility of ALND based on lymphatic drainage from the breast.

## Methods

### Study cohort

The prospective observational study enrolled consecutive breast cancer patients from November 2017 to March 2018 in a single tertiary referral academic medical center, Zhongnan Hospital of Wuhan University. Women patients meeting the following criteria and undergoing ALND were included in the study: i) patients with clinically node-positive breast cancer, defined as positive on preoperative axillary palpation, ultrasound examinations or CT scans with contrast; ii) patients who underwent mastectomy with a positive SLN; iii) patients who underwent breast-conserving surgery (BCS) for more than two positive SLNs; iv) patients who did not receive neoadjuvant chemotherapy (NCT); v) patients with no previous history of breast cancer. Those who had benign tumors and those who did not meet the above criteria were excluded from the study. The Medical Ethics Committee of Zhongnan Hospital of Wuhan University approved the routine use of the SLNB procedure for all patients. The trial was registered at the Chinese Clinical Trial Registry (ChiCTR1800014247). All patients provided written informed consent regarding SLNB and ALND.

### Dual tracers for SLNB

All patients underwent quadrantectomy or mastectomy immediately followed by SLNB. Radioactive tracers were unavailable in China. Instead, dual tracers, 1 ml (1 mg) indocyanine green (ICG) (H20045514; Weicai Pharmaceutical Corporation, Liaoning, China) and 0.5 ml (5 mg) methylene blue (MB) (H32024827; Jichuan Pharmaceutical Corporation, Taixin, China) were administered for SLNB [9].

### Surgical techniques

Before removal, all the identified SLNs were routinely and meticulously injected with 0.1–0.2 ml MB using a 1-cc syringe with a 32-gauge needle, which was called ‘staged tracing’ (Supplement 1. MP4). The careful reinjection of MB was a key procedure to not artifactually alter the lymphatic drainage patterns. MB could then flow from the SLNs along several ascending lymphatic channels towards the subclavian lymph nodes. Then, the blue-stained lymphatic channels were mapped by bluntly dissecting along the lymphatic drainage channels from the breast to the axilla. After identifying the efferent and echelon nodes, the SLNs were ready to be harvested and sent for immediate frozen sectioning (FS). Once the SLNs were confirmed positive in patients who underwent mastectomy, ALND was performed with complete resection of at least Berg’s levels I and II; the resection of Berg’s level III was performed only in patients with gross disease in Berg’s level II and/or III. When patients with BCS had more than two positive SLNs, ALND was subsequently performed.


**Additional file 1: Supplement 1.** The staged tracing procedure where 0.1 ml methylene blue is injected into the sentinel nodes

**Fig. 1:**
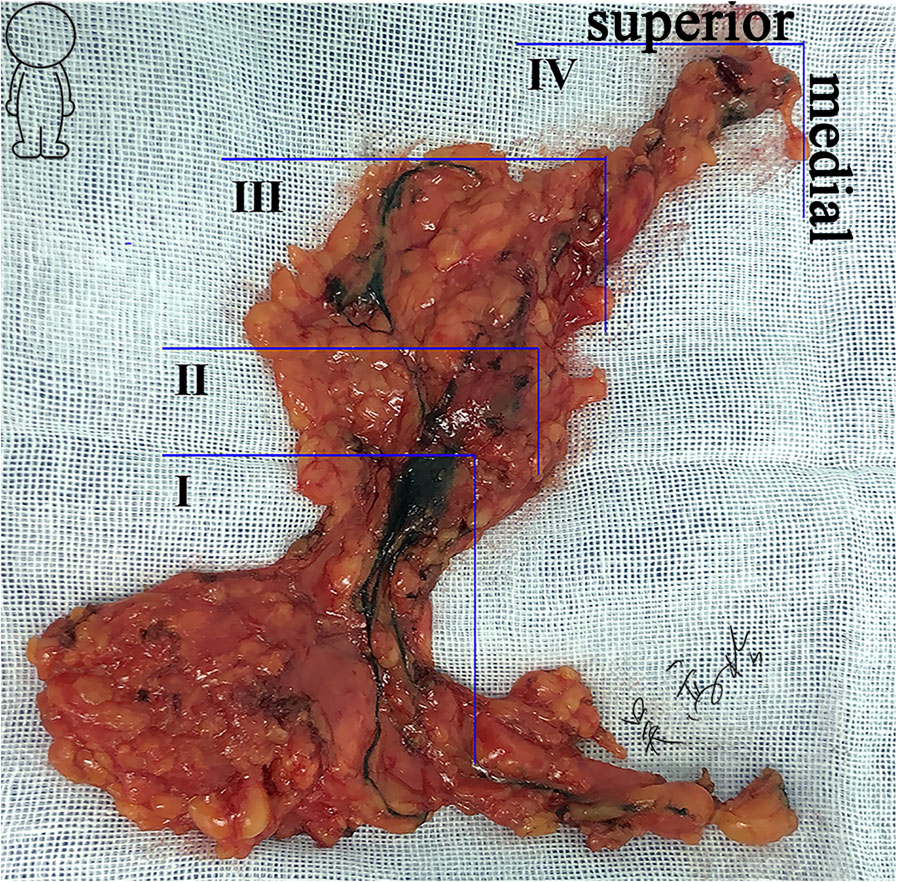
Breast lymphatics level in this study (ex vivo, right axilla)

**Fig. 2:**
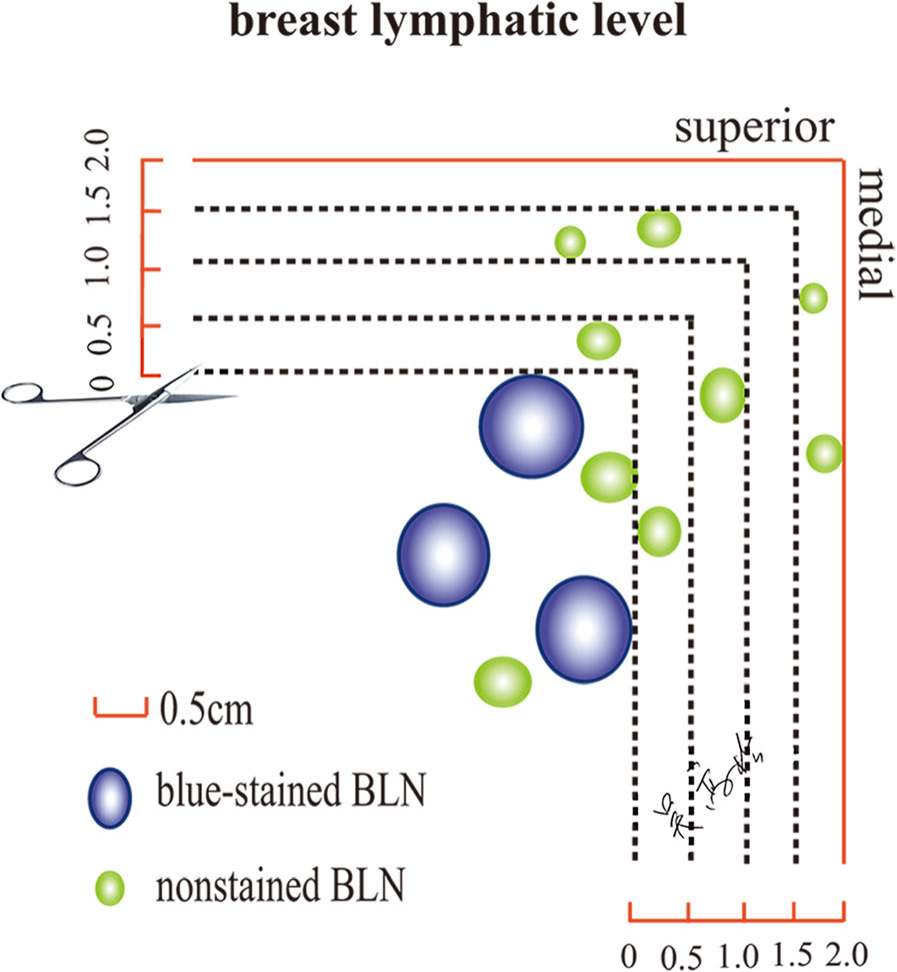
Pathological examination method (right axilla). The nonstained bALNs are sent for pathological examination separately by resecting serial tissue every 0.5 cm away from the horizontal line and vertical line along the superior blue-stained bALN and medial blue-stained bALN. bALN: axillary lymph node from the breast

After complete ALND, the removed specimen was dissected carefully ex vivo (Fig. 1). The lymph nodes adjacent to the blue-stained bALNs were defined as nonstained bALNs (Fig. 2). A horizontal line along the superior blue-stained bALN and a vertical line along the medial blue-stained bALN formed a lower outer quadrant (LOQ) zone in the axilla, which was defined as the BLL (Fig. 2). The blue-stained lymphatic channels of the breast could converge towards a group of three to five lymph nodes, namely, SLNs, at BLL I. From these nodes, there was a predictable passage of efferent lymphatics towards nodes at BLL II and, in turn, nodes at BLL III and IV (Fig. 1). With efferent lymphatics, each BLL could be distinguished. The skip metastasis was defined as macrometastatic nodes that were found at a further site while the lymph nodes nearer to the primary breast tumor were negative, as it was assumed that nodal involvement occurred in a progressive manner.

### Pathological examination

The removed SLNs were sent for immediate FS during the operation. After the operation, the nonstained bALNs were sent for pathological examination separately by resecting serial tissue every 0.5 cm away from the horizontal line and vertical line along the marginal blue-stained bALN (Fig. 2) [10]. All the retrieved bALNs were processed for routine hematoxylin and eosin staining for histology and immunohistochemically. The diameter, estrogen receptor status, progesterone receptor status and human epidermal growth factor receptor-2 status of the primary tumor were also assessed as part of a routine pathology examination.

### Comparing ALND based on BLL with standard ALND

Patients with clinical T_1–3_ and node-positive axilla were eligible and randomized to ALND based on BLL and standard ALND. Descriptive statistics were used to describe the results of the pilot prospective study. The primary objective of the pilot phase of the randomized controlled trial was to demonstrate feasibility of the procedure of ALND based on BLL trial design, and to determine if axillary recurrence rates for patients randomized to ALND based on BLL are equivalent to axillary recurrence rates for patients randomized to standard ALND.

### Statistical analysis

The demographic characteristics, tumor sizes, and number of lymph nodes in each BLL were collected. Continuous variables between groups were compared using a nonparametric test. Chi-squared tests or Fisher’s exact tests were run to compare the positive rate between the two groups. Two-sided *p* values< 0.05 were considered statistically significant. All statistical analyses were performed using SPSS17.0 for Windows (SPSS, Inc., Chicago, IL).

## Results

### Patients

A total of 190 patients underwent SLNB with dual tracers. Of these patients, 136 had node-positive axilla, and 54 patients had node-negative axilla. Two patients who failed to have any SLNs identified, 26 patients who underwent mastectomy with negative SLNs, and 6 patients who underwent BCS and planned whole-breast irradiation with fewer than 3 macrometastastic SLNs were excluded. Of the 26 patients with negative SLN identified by FS, two (7.7%) false negative cases with one lymph node were found to have macrometastatic (2 mm) disease after the postoperative H&E examination. A total of 156 patients were entered into the study, and 78.8% of the patients underwent mastectomy. Systemic therapy was recommended according to the clinical guidelines. The baseline characteristics of the SLN+ and cN+ groups are shown in Table 1. Compared with the SLN+ group, the cN+ group displayed larger tumors (*p* < 0.001). All of the patients underwent staged tracing to reveal the subsequent efferent and echelon bALNs, and MB could flow from the SLNs along several ascending lymphatic channels towards the subclavian lymph nodes. Twenty (100%) SLN+ patients and 134 (98.5%) cN+ patients successfully had the breast lymphatic vessels and bALNs identified (Fig. 1).

**Table 1:** Characteristics of 156 breast cancer patients

	SLN+ *N* = 20	cN+ *N* = 136	*p* value
Age (Mean)	49.45	51.1	0.627^a^
Tumor size, No. (%)			< 0.001^a^
< 1 cm	4 (20.0)	6 (4.4)	
1-2 cm	14 (70.0)	38 (27.9)	
2-3 cm	2 (10.0)	59 (43.4)	
> 3 cm	0	33 (24.3)	
pN stage, No. (%)			0.03^a^
N0	0	15 (11.0)	
N1	15 (75.0)	67 (49.3)	
N2	5 (25.0)	45 (33.1)	
N3	0	9 (6.6)	
Surgery, No. (%)			< 0.001^b^
Mastectomy (a SLN)	9 (45.0)	114 (83.8)	
BCS (≥3 positive SLNs)	11 (55.0)	22 (16.2)	
Histological type (%)			0.882^a^
ER/PR+, HER2-	12 (60.0)	79 (58.1)	
ER/PR+, HER2+	2 (10.0)	11 (8.1)	
ER-, PR-, HER2+	3 (15.0)	22 (16.2)	
ER-, PR-, HER2-	3 (15.0)	24 (17.6)	
Ki-67, No. (%)			0.699^a^
≥ 14%	11 (55.0)	81 (59.6)	
< 14%	9 (45.0)	55 (40.4)	
Lymphovascular invasion, No. (%)	11 (55.0)	83 (61.0)	0.607^a^
Chemotherapy, No. (%)			0.076^a^
Yes	16 (80.0)	292 (66.7)	
No	4 (20.0)	74 (33.8)	
Hormonal therapy, No. (%)			0.903^a^
Yes	14 (70.0)	97 (71.3)	
No	6 (30.0)	39 (28.7)	
Anti-HER2 therapy, No. (%)			1.000
Yes	5 (25.0)	33 (24.3)	
No	15 (75.0)	103 (75.7)	
Radiotherapy, No. (%)			0.248^a^
Yes	20 (100.0)	121 (89.0)	
No	0 (0)	15 (11.0)	
Identification rate of breast lymphatics level, No. (%)	20 (100)	134 (98.5)	1.000
Staged tracing^c^, No. (%)	17 (85.0)	91 (66.9)	0.102^b^

*SLN+* positive sentinel lymph node, *cN+* clinically node-positive axilla, *BCS* breast-conserving surgery

^a^Nonparametric test; ^b^Chi-squire test

^c^Staged tracing: injecting 0.1 ml blue dye into SLNs

Supraclavicular and infraclavicular radiotherapy was performed for patients with one or more positive lymph nodes. Chest wall radiotherapy was performed for patients who underwent BCS. 20 (100%) SLN+ patients and 121 (89.0%) cN+ patients underwent radiotherapy. The dose was 25 fractions of 2 Gy.

### Breast lymphatics level

**Fig. 3:**
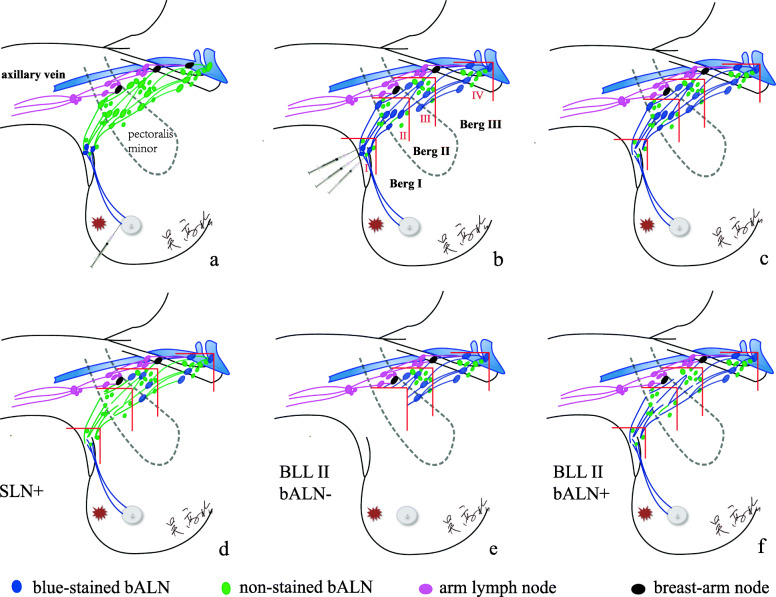
Procedures of axillary lymph node dissection based on breast lymphatics level. **a** Identify the SLNs with MB. **b** Meticulously inject 0.1–0.2 ml MB to all the SLNs. **c** SLNs were removed and sent for pathological examination by frozen section. **d** If SLN contained macrometastasis, the blue-stained bALNs in BLL II were resected and examined; **e** If the blue-stained bALNs in BLL II were confirmed negative, the tissues in BLL II were resected ‘en bloc’. **f** If the blue-stained bALNs in BLL II confirmed positive, the blue-stained bALNs in BLL III were resected and examined. SLN: sentinel lymph node; MB: methylene blue; bALNs: axillary lymph node from the breast. BLL: breast lymphatics level

The median number of BLL that could be classified was four, ranging from three to six (Fig. 1). The mean number of lymph nodes in each BLL is presented in Table 2. The skip metastasis rate of different serial distances from the marginal blue-stained bALNs is described in Table 3. While the cancer cells were not found in the blue-stained bALNs, the additional skip macrometastatic lymph nodes were located within 1.0 cm distance away from the blue-stained bALNs. No additional macrometastatic nodes were identified beyond 1.0 cm away from the marginal bALNs (Table 3). Therefore, a horizontal line 1.0 cm away from the superior blue-stained bALN and a vertical line 1.0 cm away from the medial blue-stained bALN formed a LOQ zone in the axilla, which was defined as BLL II, III, and IV (Fig. 3b). The skip metastasis rate of SLN was 9.6%, which was higher than the subsequent lymph nodes (Table 3). And no additional macrometastatic nodes were identified beyond 1.5 cm away from the marginal blue-stained SLNs. Thus, a horizontal line 1.5 cm away from the superior blue-stained SLN and a vertical line 1.5 cm away from the medial blue-stained SLN formed a LOQ zone in the axilla, which was defined as BLL I [10].

**Table 2:** Number of nodes obtained, blue-stained bALNs, and positive nodes for each breast lymphatics level

Breast lymphatics level^a^	No. of nodes obtained^b^	No. of blue-stained bALNs	No. positive nodes
Level I	5.0 (3–8)	3.5 (3–5)	3.0 (0–8)
Level II	6.5 (4–8)	5.5 (3–7)	1.0 (0–8)
Level III	5.5 (4–8)	5.0 (2–7)	0.8 (0–8)
Level IV	3.0 (2–5)	2.0 (1–3)	0.3 (0–2)
Total	19.5 (13–30)	16.5 (9–20)	5.1 (0–20)

^a^Median breast lymphatics level was four, level V and level VI were not listed

^b^No. of nodes obtained: blue-stained axillary lymph nodes from the breast (bALNs) plus non-stained bALNs, mean (mix- max)

**Table 3:** Skip metastasis rate of different serial distances from marginal blue-stained bALNs

Breast lymphatics level	Skip metastasis rate (No.)				
	0 cm^a^	0.5 cm	1.0 cm	1.5 cm	2.0 cm
level I	9.6% (15)	7.7% (12)	3.8% (6)	0	0
level II	6.5% (10)	3.9% (6)	0	0	0
level III	4.5% (7)	1.3% (2)	0	0	0
level IV	3.9% (6)	0.6% (1)	0	0	0

^a^Distance from visualized bALNs. bALN: breast lymph node

The location of the BLL was dynamic and inconsistent. The BLL I was lateral and inferior to the pectoralis minor muscle; BLL II was either located lateral and inferior to the pectoralis minor, behind the pectoralis minor, or both. In addition, BLL III was located behind the pectoralis minor, medial and superior the pectoralis minor, or both; only later BLL IV lymph nodes were medial and superior to the muscle.

### Pathology

Ninety-seven (62.2%) of the 156 patients harbored fewer than four macrometastatic lymph nodes. cN+ patients were more prone to have gross metastasis than SLN+ patients (*p* < 0.001). The distribution of the positive nodes in the BLL between the SLN+ and cN+ groups was compared and can be seen in Table 4. The cancer cells of 85.8% (121/141) of the patients with node-positive axilla metastasized from the primary tumor to BLL II.

**Table 4:** The distribution of the positive nodes in the breast lymphatics level between the two groups

Breast lymphatics level, No. (%)	SLN+ (*n* = 20)	cN+ (*n* = 121)	Total (*n* = 141)
level I	15 (75)	67 (55.4)	82 (58.1)
level II	4 (20)	35 (28.9)	39 (27.7)
level III	1 (5)	14 (11.6)	15 (10.6)
level IV	0	5 (4.1)	5 (3.5)

*SLN+* positive sentinel lymph node, *cN+* clinically node-positive axilla

### Outcome of the pilot study

The surgical procedure of ALND based on BLL was performed as follows: (1) Perform SLNB with 0.5 ml MB (Fig. 3a). (2) Routinely and meticulously inject all the identified SLNs with 0.1–0.2 ml of MB before removal (Fig. 3b). (3) After the echelon nodes were identified with injection of MB, SLNs would be harvested and pathologically examined by immediate FS (Fig. 3c). (4) If SLNs were positive, the blue-stained bALNs in BLL 2nd would be removed and sent for immediate FS (Fig. 3d). (5) If the blue-stained bALNs in BLL 2nd were confirmed negative, the tissues in BLL 2nd were resected ‘en bloc’ (Fig. 3e); (6) If the blue-stained bALNs in BLL 2nd were macrometastic, the blue-stained bALNs in lymphatic level 3rd would be removed and sent for immediate FS (Fig. 3f). Finally, the limited axillary dissection would be performed upwards from the lowest BLL that contained the first confirmed negative blue-stained bALNs, and the tissues in the matched LOQ zone would be resected en bloc.

Twenty-seven patients were enrolled in the pilot study from March 2018 to July 2018. Fourteen participants were randomized to ALND based on BLL (study group), 13 subjects were randomized to standard ALND (controlled group), and two patients withdrew from the trial. Thirteen patients from study group, and 11 patients from controlled group completed the study interventions and are included in the analysis. The median age in study group was 48.0 (range 39–62 years) and the median age in controlled group was 46.0 (range 31–61). The two groups are well-matched in terms of patient age, tumor size, biomarker profile and length of follow-up (Table 5). In the ALND based on BLL group, one (1.3%) false negative case with two lymph nodes were found to have micrometastatic (1 mm) disease after the postoperative H&E examination. After a median follow-up of 24 months (range 22–25 months), no axillary recurrence and distant metastasis event was observed in the ALND based on BLL group and standard ALND group. One (7.7%) patients in the ALND based on BLL group and two (18.2%) patients in the standard group occurred arm lymphedema (Table 5).

**Table 5:** Baseline characteristics of the two group participants in the pilot study

	ALND based on BLL *N* = 13	Standard ALND *N* = 11	*p* value
Patients, No. (%)			0.478
SLN+	4 (30.8)	2 (18.2)	
cN+	9 (69.2)	9 (81.8)	
Age, Median (min, max)	48.0 (39, 62)	46.0 (31, 61)	0.239b
BMI, Mean (min, max)	24.1 (18.4, 28.4)	25.4 (19.1, 28.3)	0.364b
No. of nodes removed, mean (min, max)	8.0 (7, 17)	11.0 (10, 18)	< 0.001b
Tumor size, n (%)			0.886^a^
T1b/a/mi	1 (7.7)	1 (9.1)	
T1c	4 (19.2)	3 (27.3)	
T2	7 (53.8)	5 (45.5)	
T3	1 (7.7)	2 (18.2)	
Pathological nodal status, No. (%)			0.531^a^
N_0_	0	0	
N_1_	7 (53.8)	5 (45.5)	
N_2_	6 (46.2)	5 (45.5)	
N_3_	0	1 (9.1)	
Tumor grade, No. (%)			0.850^a^
I	2 (15.4)	2 (18.2)	
II a	4 (30.8)	3 (27.3)	
II b	3 (23.1)	4 (36.4)	
III	4 (30.8)	2 (18.2)	
Tumor subtype, No. (%)			0.967^a^
ER/PR+, HER2-	6 (46.2)	5 (45.5)	
ER/PR+, HER2+	2 (15.4)	2 (18.2)	
ER-, PR-, HER2+	3 (23.1)	3 (27.3)	
ER-, PR-, HER2-	2 (15.4)	1 (9.1)	
Ki-67, No. (%)			0.973^a^
≥ 14%	7 (53.8)	6 (54.5)	
< 14%	6 (46.2)	5 (45.5)	
Lymphovascular invasion, No. (%)	8 (61.5)	7 (63.6)	1.000
Chemotherapy, No. (%)			0.834^a^
Yes	11 (84.6)	8 (72.7)	
No	2 (15.4)	3 (27.3)	
Hormonal therapy, No. (%)			1.000
Yes	8 (61.5)	7 (63.6)	
No	5 (38.5)	4 (36.4)	
Anti-HER2 therapy, No. (%)			1.000
Yes	5 (38.5)	5 (45.5)	
No	8 (61.5)	6 (54.5)	
Radiotherapy, No. (%)			–
Yes	13 (100.0)	11 (100.0)	
No	0 (0)	0 (0)	
Arm lymphedema, No. (%)	1 (7.7)	2 (18.2)	0.877^a^
Locoregional recurrence, No. (%)	0 (0)	0 (0)	–

*cN+* clinically node-positive axilla, *SLN+* positive sentinel lymph ndoe, *ALND* axillary lymph node dissection, *BLL* breast lymphatics level, *BMI* body mass index, *HER2* human epidermal growth factor receptor 2

^a^Chi-square test, bnonparametric test

## Discussion

In this prospective observational study, ALND based on BLL attempted to resect potentially metastatic tissues level by level to minimize the extent and morbidity of ALND. Staged tracing (injection of 0.1 ml MB into the SLNs) was utilized to reveal the breast lymphatic system in the axilla basin. The median number of BLL was four, ranging from three to six. A horizontal line 1.0 cm away from the superior blue-stained bALN and a vertical line 1.0 cm away from the medial blue-stained bALN formed BLL II, III, IV. The skip metastasis rate was zero when an en bloc resection was performed upwards towards the BLL that contained the first confirmed negative blue-stained bALN. As described in a previous study, a horizontal line 1.5 cm away from the superior blue-stained bALN and a vertical line 1.5 cm away from the medial blue-stained bALN formed BLL I, which was proposed to be removed en bloc in breast cancer patients with negative SLN to reduce the number of false-negative events from SLNB [10]. In the present study, through resecting the lymph nodes level by level for breast cancer patients with node-positive axilla, the surgical approach of ALND based on BLL was valuable in reducing the BCRL rate without reducing cancer control.

Depending on the various criteria of BCRL and the extent of axillary dissection, a pooled estimation of the arm lymphedema rate is 16.6% (95% CI 13.6–20.2) [11]. The risk factors of BCRL can be affected by two aspects: demographic and lifestyle [11], and breast cancer-related variables, including radiotherapy to the axilla, number of nodes involved and removed, and taxane-based chemotherapy. In addition, a hypothesis was proposed that the transection of lymphatic vessels that drain the arm during their course through the axilla during complete ALND was associated with BCRL [12]. Thompson et al. [13] and Nos et al. [14]. have previously described a new technique, axillary reverse mapping (ARM), to identify and preserve arm lymph nodes, which reduced the number of arm lymphedema events [15]. A refined ARM technique was proposed in our institution to identify the arm lymphatic system and eliminate postoperative arm lymphedema [16, 17]. An intact pathway for lymphatic arm drainage is adjacent to the axillary vein and is usually located above the second intercostobrachial nerve. Hence, a horizontal line in this study was designed as the upper landmark during ALND surgery based on BLL to protect the arm lymphatic system. In addition, according to the direction of the lymphatic drainage, the medial and superior blue-stained bALNs were selected as the landmark.

Over the past few years, axillary management has changed greatly [18]. With effective multidisciplinary treatment, the theory of breast cancer surgery leans towards “less is more” [19]. After the publication of the ACOSOG-Z0011 and AMAROS trials, varieties of patterns of care for axillary surgery were present [20], particularly for cT_1-2_N_0_M_0_ patients with positive SLNs, which aimed to decrease the treatment-related morbidity without reducing cancer control. For breast cancer patients with cN+ axilla, NCT was often performed and targeted axillary dissection was done to identify the patients who might not require ALND [21]. The omission of complete ALND in these studies was associated with much lower rates of lymphedema. However, clinically node-positive patients who undergo ALND and patients who are not eligible based on the Z0011 criteria also need de-escalate surgical areas for ALND. Approximately 25% of the patients who undergo SLNB have positive nodes, and these patients undergo ALND and remain at risk for arm lymphedema [22].

As is well-known, metastasis from breast cancer does not involve the breast regional lymph nodes as a unit but rather progresses from the primary tumor to the first-line draining nodes and, in turn, sequentially to the second and third echelon nodes [23]. Based on the biological and anatomical rationale of SLNB, the approach of ALND based on BLL was proposed in our institution to balance the demand of preventing axillary recurrence and the wish of avoiding treatment-related morbidity, particularly arm lymphedema. Classifying the bALNs according to lymphatic drainage is a feasible and dynamic way to limit axillary surgical dissection. In the present study, to dispel skip metastasis, nonstained bALNs were sent for pathological examination separately by resecting serial tissue every 0.5 cm away from the horizontal line and vertical line along the marginal blue-stained bALN (Fig. 2). In cases of skip metastasis, additional involved nodes were found within the area 1.0 cm away from the marginal blue-stained bALNs (Table 3). Therefore, a horizontal line 1.0 cm away from the superior blue-stained bALN and a vertical line 1.0 cm away from the medial blue-stained bALN formed the BLL II, III, IV. The skip metastasis rate was zero when en bloc resection was performed upwards towards the BLL that contained the first confirmed negative blue-stained bALN (Fig. 3f), which could limit the extent of axillary dissection and reduce the number of BCRL events.

Potential limitations existed in the present study. Considering that NCT could influence the structure of the breast lymphatics and lead to an incomplete lymphatic pathway, patients who underwent NCT were excluded from the study. The pilot phase of the randomized controlled trial comparing ALND based on BLL and standard ALND revealed a satisfactory outcome. Further randomized controlled trial was needed to confirm its effect.

## Conclusion

With the ALND based on BLL approach, a more focused and less radical axillary dissection to remove the disease can be performed. To determine the precise scope of the axillary dissection, a horizontal line 1.0 cm away from the superior blue-stained bALN and a vertical line 1.0 cm away from the medial blue-stained bALN formed an LOQ zone in the axilla, which was defined as BLL II, III, IV. This new classification of the breast lymphatics could minimize the axillary dissection for breast cancer patients with pathological node-positive axilla and the potential to reduce BCRL events.

## Data Availability

Due to the privacy of patients, the data related to patients cannot be available for public access but can be obtained from the corresponding author on reasonable request approved by the institutional review board of Wuhan University of Zhongnan Hospital. (wugaosong@whu.edu.cn).

## Abbreviations

SLNB: Sentinel lymph node biopsy
ALND: Axillary lymph node dissection
cN+: clinically node-positive axilla
BCRL: Breast cancer-related lymphedema
SLN: Sentinel lymph node
bALN: axillary lymph node from the breast
BLL: Breast lymphatics level
NCT: Neoadjuvant chemotherapy
ICG: Indocyanine green
MB: Methylene blue
FS: Frozen sectioning
LOQ: Lower outer quadrant
ARM: Axillary reverse mapping
CARE: Conservative axillary regional excision
